# The Impact of COVID-19 on the Guillain–Barré Syndrome Incidence

**DOI:** 10.3390/biomedicines12061248

**Published:** 2024-06-04

**Authors:** Magdalena Kopańko, Magdalena Zabłudowska, Monika Zajkowska, Monika Gudowska-Sawczuk, Mateusz Mucha, Barbara Mroczko

**Affiliations:** 1 Department of Biochemical Diagnostics, Medical University of Bialystok, 15-269 Bialystok, Polandmonika.gudowska-sawczuk@umb.edu.pl (M.G.-S.); mroczko@umb.edu.pl (B.M.); 2 Department of Neurodegeneration Diagnostics, Medical University of Bialystok, 15-269 Bialystok, Poland; 3 Department of Oncological Surgery with Specialized Cancer Treatment Units, Maria Sklodowska-Curie Oncology Center, 15-027 Bialystok, Poland

**Keywords:** SARS-CoV-2, COVID-19, viral infection, neurological disorder, Guillain–Barré syndrome

## Abstract

Despite the fact that the global COVID-19 pandemic has officially ended, we continue to feel its effects and discover new correlations between SARS-CoV-2 infection and changes in the organism that have occurred in patients. It has been shown that the disease can be associated with a variety of complications, including disorders of the nervous system such as a characteristic loss of smell and taste, as well as less commonly reported incidents such as cranial polyneuropathy or neuromuscular disorders. Nervous system diseases that are suspected to be related to COVID-19 include Guillain–Barré syndrome, which is frequently caused by viruses. During the course of the disease, autoimmunity destroys peripheral nerves, which despite its rare occurrence, can lead to serious consequences, such as symmetrical muscle weakness and deep reflexes, or even their complete abolition. Since the beginning of the pandemic, case reports suggesting a relationship between these two disease entities have been published, and in some countries, the increasing number of Guillain–Barré syndrome cases have also been reported. This suggests that previous contact with SARS-CoV-2 may have had an impact on their occurrence. This article is a review and summary of the literature that raises awareness of the neurological symptoms’ prevalence, including Guillain–Barré syndrome, which may be impacted by the commonly occurring COVID-19 disease or vaccination against it. The aim of this review was to better understand the mechanisms of the virus’s action on the nervous system, allowing for better detection and the prevention of its complications.

## 1. Introduction

SARS-CoV-2 (severe acute respiratory syndrome coronavirus 2) is a widely known virus that causes an acute respiratory disease called coronavirus disease 2019 (COVID-19) [1,2]. The virus was first discovered in Wuhan (China) in December 2019 and assigned to the coronavirus family, a beta subfamily of single-stranded positive RNA (+ssRNA). Its genome is approximately 30 kb, and the four most important structural proteins it encodes are the nucleocapsid, membrane, envelope, and spike proteins [3,4]. Infection causes a variety of respiratory and non-respiratory symptoms (e.g., gastrointestinal, hepatic, renal, cardiac, or hematological symptoms) [5,6], of which the most common of them include cough, shortness of breath, fever, muscle pain, diarrhea, or lymphopenia observed in laboratory tests [7,8,9]. Although it primarily causes respiratory tract infections, patients have also reported various neurological symptoms, such as headaches, dizziness, confusion, and a characteristic loss of smell and taste [7,10]. There have been numerous cases of neurological diseases suspected to be caused by the virus, such as meningitis and encephalopathy [11,12]. Since the beginning of the pandemic, there have been numerous reports of Guillain–Barré syndrome, which is suspected to be related to the SARS-CoV-2 infection [13,14,15]. Symptoms of this syndrome are also increasingly reported following vaccination against coronavirus [16,17].

Guillain–Barré syndrome (GBS) is an acute, inflammatory autoimmune disease of the nervous system characterized by the destruction of peripheral nerve myelin sheaths, damage to axons, or both [18,19]. The most common symptoms of GBS, despite the rarity of the syndrome, are severe and include symmetrical and progressive motor paralysis and loss of reflexes, which may develop within a few days or even hours [20]. The occurrence of GBS associated with COVID-19 infection or vaccination against the virus affects different variants of this syndrome. Cases of patients diagnosed with acute inflammatory demyelinating polyneuropathy (AIDP), in which Schwann cells and the myelin sheath on sensory and motor nerves are damaged after vaccination against COVID-19 were examined. Patients in this group were characterized by general muscle weakness, upper-limb weakness, lower-limb weakness, quadriparesis, paraplegia, ascending paralysis, numbness, diarrhea, Bell’s palsy, and dysphagia [21,22,23]. There have been cases of hospitalized patients diagnosed with motor axonal neuropathy (AMAN) and acute motor and sensory axonal neuropathy (AMSAN) after AstraZeneca vaccination, which is caused by the presence of autoantibodies against gangliosides, components of the axonal membrane. They experienced neurological symptoms, such as tetraplegia, cranial nerve involvement, facial diplegia, sensory involvement, bulbar symptoms, facial diplegia with ophthalmoplegia, and paraparesis [21,22,24]. There are even reports of rare Miller-Fisher syndrome (MFS) after COVID-19 infection. The case of a man who developed weakness of the upper and lower extremities, and weakness of the facial muscles, ptosis, dysphagia, and paraesthesia have been described [18,21,22,25,26].

The incidence of Guillain–Barré syndrome is approximately 1 case per 100,000 people annually, with over 20% of cases being serious, general forms of the disease, leading to respiratory failure. Susceptibility to the disease increases with age. Men are more likely to be affected by the disease than women. In order to alleviate the effects of the disease, in addition to supportive treatment, immunoglobulins are administered intravenously, or plasmapheresis is performed [27]. The factors causing GBS are usually viral infections (e.g., Zika virus or EBV; cases of GBS have also been observed in isolated patients with active-infection MERS-CoV, which is genetically similar to SARS-CoV-2). Demyelinating polyradiculoneuropathy can also be caused by bacterial infections (e.g., *Campylobacter jejuni, Mycoplasma pneumoniae*, or *Haemophilus influenzae*) [28,29,30,31] which can originate the appearance of GBS symptoms, usually within 1–4 weeks after infection [18]. Cases of this syndrome have also been reported after taking vaccines, e.g., against the hepatitis B virus or influenza. However, there was no statistically significant increase in the risk of GBS after vaccination [32]. The disease also occurred after surgical procedures or as a result of developing cancer [27,33]. Unfortunately, despite treatment, about 9–17% of GBS cases result in disability or even the death of a patient [34].

This review article is a summary of information about the potential impact of SARS-CoV-2 on the nervous system, as well as the available data on reported cases of GBS following infection caused by SARS-CoV-2 virus or as a result of vaccination against COVID-19, which may serve as the foundation for further investigation into the relationships between these two variables.

## 2. Neurological Symptoms of SARS-CoV-2 Infection

In addition to typical respiratory symptoms, patients infected with the SARS-CoV-2 virus may also experience symptoms from the nervous system. Research performed so far has revealed that patients with COVID-19 had a higher risk of possible neurological and psychiatric complications than patients with influenza or other respiratory diseases [35]. Additionally, compared to those with mild infections, patients with serious infections are more likely predisposed to develop neurological disorders [36]. According to Yachou et al., the neurological symptoms caused by SARS-CoV-2 are the result of the presence of a massive excess of pro-inflammatory cytokines and, associated with it, subsequent cytokine storm [37]. Interestingly, it has been shown that people who previously suffered from chronic neurological diseases are characterized by higher mortality rates from COVID-19 than neurologically healthy individuals [38].

There have been reported many cases of peripheral nervous system (PNS) diseases suspected to be related to COVID-19. The virus attacks the peripheral nervous system (PNS) in the form of peripheral polyneuropathy (PNP), e.g., cranial polyneuropathy, which, in this case, is manifested by facial palsy and olfactory disturbance [39]. Probably in April 2020, the information concerning the first neurosensory hearing loss in Thailand was published. The case study concerned a female who was old, while in the country, the total number of cases was only 82 [40]. COVID-19 has been linked to a less-known Parsonage–Turner syndrome, usually manifested by sudden severe shoulder pain followed by motor weakness [41] or with an appearance later neuromuscular disorders [42]. In addition, myasthenia gravis can be included in the neuromuscular junction disorders that occurred in three patients without previous neurological or autoimmune disorders [43]. According to Mao et al., more than 35% of the 214 patients hospitalized in connection with COVID-19 developed neurological symptoms [36]. According to findings by Herrero-Montes et al., after recovering from the illness, about 25% of hospitalized survivors experienced neuropathic pain [44]. As shown by the above-collected information about the symptoms of infection or complications of COVID-19 regarding the peripheral nervous system were very diverse, and some of them occurred in a significant number of cases, which shows that the coronavirus has a major impact on the nervous system.

## 3. Pathomechanism of Guillain–Barré Syndrome Associated with SARS-CoV-2

The diagnosis of GBS consists of a medical interview, followed by electrophysiological, neurological, and CSF examinations [45]. The pathogenesis of GBS associated with COVID-19 is not fully understood, but probable mechanisms have been described in the literature.

**Figure 1:**
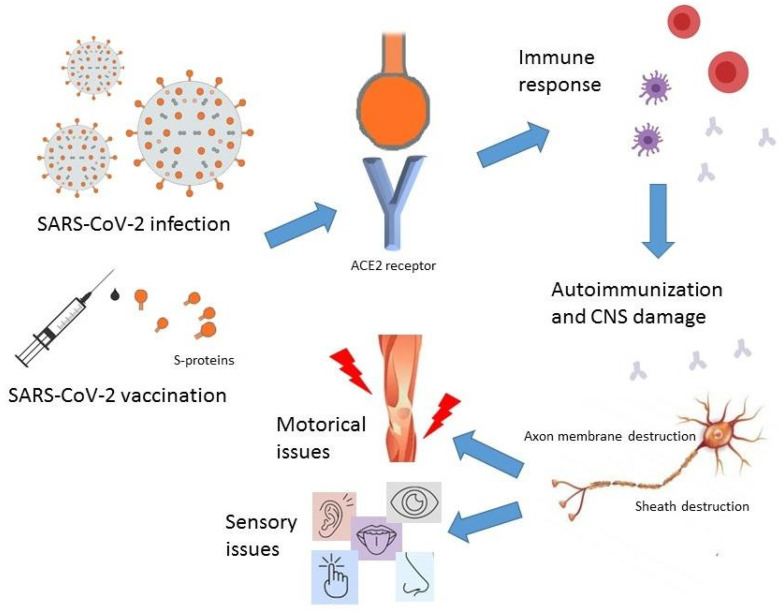
Mechanism of SARS-CoV-2 action in GBS.

The probable mechanism of autoimmunity and the occurrence of Guillain–Barré syndrome is the appearance of molecular mimicry [46], which involves the induction of myelin autoimmunity as a result of an infection by a microorganism having epitopes resembling the structures of the myelin sheath [47]. The infection causes sensitization of cross-reacting T cells and the production of antibodies against myelin components, which cause demyelination and conduction block. The synthesis of autoantibodies directed against peripheral nerve elements, and especially against gangliosides, plays a significant role in the pathogenesis of Guillain–Barré syndrome [46,48]. It has been suggested that the bonding of the SARS-CoV-2 S protein with ACE2 receptors or gangliosides containing sialic acid residues may lead to the synthesis of autoantibodies directed against those targets (Figure 1) [49]. This is consistent with the data obtained by Pimentel et al., who found that the most prevalent GBS antibodies in the course of COVID-19 disease are anti-GD1b antibodies [50]. However, it has been demonstrated that antibodies directed against gangliosides develop in a relatively small subset of patients (according to various analyses, from only a few to approximately 20%) following COVID-19 [50,51,52].

Comparing the pre-pandemic period with the pandemic, the number of positive GQ1b and G1M antibody test results was significantly lower during the pandemic, but the anti-GD1a and anti-GD1b antibody positive indicators were similar. This study was conducted even before the release of the vaccination against SARS-CoV-2 [53]. 

Homeostasis of the nervous system is maintained by the presence of barriers that separate it from the blood and external factors. The blood–nerve barrier (BNB) is a protective barrier of the peripheral nervous system composed of intraneural microvessels, specifically the endothelium, that, under physiological conditions, strictly controls the flow of substances and leukocytes between the blood and the intranerve [54]. The damage or dysfunction of BNB is one of the features of GBS [55]. 

According to Gao et al.’s analysis, NLR (NOD-like receptor) and TLR (Toll-like receptor) signaling pathways play a significant role in the relationship between COVID-19 and GBS. They are involved in detecting the virus and the activation of the proinflammatory cytokines synthesis, which significantly contribute to the development of GBS [56]. PCR tests on the cerebrospinal fluid of patients for the presence of the COVID-19 virus were negative in the majority of cases, indicating that the most common is the immunological, post-infective mechanism of viral invasion, rather than a direct (parainfection) mechanism [52].

## 4. COVID-19 and Guillain–Barre Association

The first case of GBS associated with COVID-19 infection was documented in January 2020. It concerned a 61-year-old woman who initially did not show the typical symptoms of infection, but very rapid symmetrical weakness and areflexia of both legs, feet, and arms, as well as the hand muscles, were noted [14]. Since the pandemic began, there have been numerous cases of GBS following the virus. Meta-analyses conducted so far indicate that GBS in the course of COVID-19 most often occurred in older people, especially men [50,57,58,59]. However, these two factors were also general risk factors for COVID-19 [60] and mortality due to this disease [61]. Nonetheless, cases of GBS associated with COVID-19 in children have also been described [59,62,63,64,65].

In some parts of the world, such as Northern Italy, an increase in GBS cases was observed in the initial months of the pandemic compared to the previous year, and it was noted that GBS after COVID-19 infection was also more severe [66]. In Spain [67] and Brazil [68], an increased frequency and severity of GBS was reported in patients with COVID-19. Conversely, the opposite results were obtained in the UK, where, according to data from the national immunoglobulin database, there was a decrease in the number of cases [69]. A similar situation was also observed in Sweden [70], Korea [71,72], and Portugal [73]. The reduction in the number of GBS cases may be due to the protective measures introduced during the pandemic. However, based on data collected in Iran, no significant difference between the incidence of GBS cases before and during the pandemic was observed [74]. According to a cohort study conducted in India, there was a pandemic reduction in the number of cases, in this case of pediatric GBS, but the disease profile remained unchanged [75]. The incidence of GBS in patients with COVID-19 was estimated at 1:62,500 [69]. Cases of GBS after COVID-19 infection affect patients of any severity of infection. However, most of them required hospital treatment, intensive care units, or mechanical ventilation during infection [14,50,76]. According to a meta-analysis by Bentley, Bentley, S.A., et al., patients were hospitalized for 23 ± 17 days. About 45% of them were accepted into intensive care units, and a significant part (38%) required mechanical ventilation [52]. The conducted analyses, comparing patients with GBS after COVID-19 with patients who have not undergone infection, showed that the symptoms of GBS are more severe in patients who have previously suffered from COVID-19 [57,66].

The most common symptoms of infection before the onset of GBS were fever, shortness of breath, cough, and gastrointestinal symptoms. Single cases of patients did not show symptoms of the virus before GBS [76]. According to a meta-analysis conducted by Samir Abu-Rumeileh et. al., the symptoms of GBS usually appeared after the symptoms of COVID-19. In single cases, they occurred before, after, or in parallel with them [77], but such cases also occurred [78]. The average time for symptoms suggestive of GBS to appear after a SARS-CoV-2 infection is approximately 10 days. However, it turns out that GBS can appear even after a longer period of latency. Kuang, Wayne, et al. described the case of a 26-year-old man whose symptoms, consistent with Miller-Fisher syndrome, occurred 10 weeks after the infection. This is also the case of the youngest adult who developed GBS after COVID-19 [26]. Based on the analysis conducted by Uncini A on a group of 42 patients who developed GBS after COVID-19, most of them (71.4%) experienced typical symptoms that classify GBS, such as symmetrical weakness of the limbs, sensory symptoms, and reduced or absent tendon reflexes. However, the most common symptoms of GBS appearing in these patients include hyporeflexia (80.9%), sensory disturbances (66.7%), limb weakness (76.2%), and facial palsy (38.1%) [78]. Hülya Ertaşoğlu Toydemir et al. showed that people with GBS after COVID-19 more often suffered from facial diplegia than patients with GBS who did not contract COVID-19 [79]. COVID-19-associated GBS typically exhibited electrophysiological features, suggesting AIDP-specific demyelination [49,50,52,80], which is the most common type of GBS and accounts for up to 90% of all GBS cases [19]. Demyelination was more advanced in GBS cases in people who had COVID-19 than in other patients [66]. In some cases, the types of GBS overlapped. The results of a meta-analysis concerned the electrophysiological assessment of the nerve condition carried out using SNAP (sensory nerve action potential) and CMAP (complex muscle action potential) tests showed abnormalities in 86.9% and 99% of patients suspected of GBS [52].

## 5. COVID-19 Vaccine-Associated GBS

Pfizer-BioNTech, Moderna, Johnson & Johnson (J&J), Sputnik, AstraZeneca, and Sinopharm are just some of the many COVID-19 vaccines available [81]. Their mechanisms of action are diverse. For example, the Pfizer and Moderna vaccines are mRNA-based vaccines. Meanwhile, J&J, Sputnik, and AstraZeneca are based on an adenoviral vector, and Sinopharm is an inactivated vaccine. However, all of them have the same purpose, which is to produce antibodies against the spike protein [82,83]. Typical side effects after receiving the vaccine most often include headache, fatigue, fever, pain at the injection site, muscle pain, or nausea [84,85,86,87]. According to a survey conducted by Renuka AK Kadali among healthcare workers, over 60% of people who completed the survey reported experiencing at least one side effect after receiving the Comirnaty mRNA Pfizer vaccine. However, most of them (almost 80%) were still able to perform everyday activities. Only 2.49% required ambulatory treatment, 0.62% a visit to the Acute Admissions Unit, and 0.25% hospitalization [86]. Interestingly, women were most likely to experience post-vaccination side effects from the nervous system [88,89]. Less-common complications include thrombotic complications and thrombocytopenia [90], Bell’s palsy [91], or *Herpes zoster* virus reactivation [92].

Waheed S. et al. presented the earliest case of Guillain–Barré syndrome after receiving one dose of the COVID-19 mRNA vaccine produced by Pfizer. It concerned an 82-year-old woman who, a week after vaccination, noticed body pain and malaise. Within 2 weeks of receiving the vaccination, she experienced difficulty walking. After tests of cerebrospinal fluid and magnetic resonance imaging of the lumbar spine, the diagnosis of GBS syndrome was confirmed [93]. The occurrence of GBS after administration of the COVID-19 vaccine is most likely caused by the induction of an autoimmune reaction leading to the production of anti-myelin antibodies as a result of the cross reaction of anti-S antibodies with gangliosides [94]. The incidence of GBS after receiving the vaccine was estimated at approximately eight per million vaccinations, of which the majority of cases included in the analysis concerned the AstraZeneca vaccine, which is one of the vaccines based on an adenoviral vector [95]. Many other analyses also noted a high incidence of GBS after receiving the AstraZeneca vaccine [96]. Similar results were obtained in France, where it was estimated that, for the first dose of the AstraZeneca vaccine, there were 6.5 cases of GBS per million people, while in the case of the Janssen adenoviral vaccine, it was 5.7 [24]. GBS symptoms were observed after the first and second doses of the vaccine [24,97,98]. What is of the utmost importance, according to the Center for Disease Control, is that adults vaccinated with the J&J vaccine (adenoviral) are also at increased risk of GBS, but this does not apply to people who received the mRNA vaccines (Pfizer-BioNTech or Moderna) [99]. Based on the analyzed data, the occurrence of GBS after the COVID-19 vaccination did not dominate in any gender or age group but was connected with type of the vaccine [24,93,94,98].

The literature describes cases of patients who developed GBS symptoms after receiving a combination of vaccinations against SARS-CoV-2 and COVID-19 infection. Aomar-Millán et al. described the case of a 77-year-old man who was admitted to the hospital due to mild lung involvement by SARS-CoV-2. Two weeks after discharge, the patient received the Pfizer COVID-19 vaccine, and after 3 days, began to experience symptoms of lower-limb weakness. Ultimately, the diagnosis of AMSAN was confirmed [100]. Bellucci et al. described an interesting case of a patient who was diagnosed with GBS associated with COVID-19 infection during the pandemic. The treatment led to the patient’s recovery. After half a year, he received the first dose of the Pfizer SARS-CoV-2 vaccine, and shortly thereafter, GBS relapsed [101]. There was also a case of a patient who, after taking the first dose of the Moderna vaccine, experienced bilateral lower extreme paresthesia, then progressive bilateral lower-limb distal weakness and problems with movement. After receiving the second dose, the subject went to emergency, where tests revealed AIDP. A year earlier, the patient suffered from COVID-19 [102]. In Vietnam, there was a case of a patient who, as a result of vaccination with Sinopharm, developed GBS, which was then followed by COVID-19 infection during hospitalization. His condition deteriorated significantly. Acute respiratory distress syndrome (ARDS) developed, and after the illness, an additional symptom of GBS occurred: peripheral facial paralysis [83]. An elderly man went to hospital 3 days after receiving the AstraZeneca vaccine due to symptoms of COVID-19 disease. The day after the breathing difficulties ceased, he suffered acute paresis of all four limbs, speech disorders, and respiratory failure occurred again, requiring intubation [100]. Despite the described cases of GBS that have been linked to vaccination and infection, we do not have enough data to determine whether vaccination with a concomitant COVID-19 infection has a stronger impact on the occurrence of GBS than a single factor or if the symptoms are more severe in one of the described cases.

Beliefs about the overall impact of vaccination against COVID-19 are not clear. There are studies suggesting that vaccination against SARS-CoV-2 reduced the number of cases of post-COVID GBS. An analysis by Finsterer J. et al. shows that the number of published cases of GBS in the second half of 2020 was higher than in the first half of 2021, so the author suggests that the number of patients suffering from GBS has also decreased [103]. According to data from VigiBase, the risk is virtually the same as with other antiviral vaccines. However, the incidence of GBS after the vaccine is lower than after COVID-19 [104]. A study conducted in Israel showed that the risk of developing GBS after the Comirnaty Pfizer vaccine was approximately 15 times lower than in the case of the SARS-CoV-2 infection [105]. According to a meta-analysis conducted by Stefano Censi et al., adenovirus-based vaccines demonstrated a 2.4-fold odds ratio of GBS, which was approximately seven times greater than that observed with mRNA vaccines. The lower incidence of GBS linked to mRNA vaccines may be attributed to their potential in reducing infections, including SARS-CoV-2 [106]. Despite the presented complications after receiving vaccines against SARS-CoV-2, it should be noted that their effectiveness (91-98% in the case of the Pfizer-BioNTech and Moderna vaccines) against COVID-19 infections and symptoms may significantly outweigh the risk of side effects, including GBS syndrome [107]. According to some authors, it definitely outweighs these risks [106]. Based on the above-mentioned data, it can be assumed that vaccines using an adenovirus vector have been described as a possible cause of GBS with a much higher frequency compared to mRNA vaccines. Table 1 summarizes all described neurological complications occurring in patients after vaccination, depending on the vaccine used. 

## 6. Conclusions

Nervous system diseases that are suspected to be related to COVID-19 include Guillain–Barré syndrome. The number of GBS cases may depend on environmental factors. However, it is suggested that the overall number of GBS patients may be reduced as a result of restrictive protective measures during the pandemic. Based on the available data, it is difficult to clearly determine whether SARS-CoV-2 infection affects the frequency of GBS, as the results vary by country. However, there are clear reports of increased severity of GBS following infection. According to the data concerning the SARS-CoV-2 vaccinations, there have been cases presented in the literature in which the authors indicated that the vaccination was the probable cause of Guillain–Barré syndrome. What is more, adenovirus-based vaccines demonstrated a much higher incidence of GBS than mRNA vaccines, which may be related to their diverse mechanism of action. However, in order to obtain more accurate and reliable data, it would be necessary to conduct well-designed studies, due to the difficulty of obtaining all the information necessary for evaluation from individual cases or meta-analyses. They could allow us to better assess the relationship between these factors and, thus, prevent at least some neurological health effects and improve the quality of life of patients. Despite reports from some countries showing no increase in GBS cases during the pandemic, it is worth considering the possibility of infection with the SARS-CoV-2 virus and the type of vaccine, if any, used when diagnosing Guillain–Barré syndrome.

## Figures and Tables

**Table 1:** Summary of neurological complications occurring in patients after vaccination, depending on the vaccine used.

Type of Vaccine	Count of GBS Cases	Associated Neurological Complications
Pfizer	1 [70] 1 [93] 28 [95] 5 [96] 4 [98] 1 [100] 1 [101] Total: 41	difficulty walking [94] shoulder pain, arm weakness and areflexia [94] numbness, swelling, pain on the left side of the face and neck [98]
AstraZeneca	15 [24] 1 [70] 77 [95] 8 [96] 14 [98] 1 [100] Total: 116	acute tingling and weakness in hands and feet, severe back and chest pain, altered taste, paresthesia in the tongue and perioral area, distal paresthesia, facial paresthesia, weakness, areflexic quadriplegia, facial diplegia and respiratory failure, paraplegia, ophthalmoplegia, bulbar symptoms, abducens palsy [93,94,97,98]
J&J	9 [93] 1 [98] Total: 10	back and leg pain, headache, double vision, facial diplegia, paraparesis of lower legs [98]
Moderna	7 [93] 2 [102]	progressive, ascending bilateral lower extremity paresthesia and weakness [102]
Sputnik	9 [93]	Data not available
Sinopharm	1 [83]	paralysis of the lower and upper extremities [83]

J&J; Johnson & Johnson.

## Data Availability

Not applicable.
